# Impact of obesity on perioperative outcomes in robot-assisted surgery for endometrial cancer: A single-center study of 119 cases

**DOI:** 10.1016/j.eurox.2026.100446

**Published:** 2026-01-31

**Authors:** Yasushi Iida, Miwako Shimazaki, Ayane Kosuge, Takahiro Matsunami, Kosuke Kato, Teppei Ichikawa, Taichi Irie, Makoto Iizuka, Daishi Hirano, Satoshi Takakura

**Affiliations:** a Department of Obstetrics and Gynecology, Dokkyo Medical University Saitama Medical Center Koshigaya, Japan; b Department of Pediatrics, The Jikei University School of Medicine, Japan

**Keywords:** Robotic surgical procedures, Endometrial cancer, Obesity, Body mass index, Length of stay, Lymph node yield

## Abstract

**Introduction:**

Whether obesity (body mass index [BMI] ≥30 kg/m²) independently affects perioperative outcomes in robot-assisted surgery for endometrial cancer remains uncertain.

**Materials and methods:**

We conducted a retrospective single-center cohort including 119 consecutive patients who underwent a uniform robotic procedure—total hysterectomy with bilateral salpingo-oophorectomy and pelvic lymphadenectomy using the da Vinci Xi system—between November 2018 and June 2025. Patients were grouped by BMI ≥ 30 (n = 32) versus < 30 (n = 87). Outcomes were estimated blood loss (EBL), operative time, lymph-node yield, and length of stay (LOS). Multivariable linear regression adjusted for age, prior abdominal/pelvic surgery, comorbidity history (diabetes, hypertension, dyslipidemia), operating surgeon, and surgical year (2018–2020 vs 2021–2025).

**Results:**

BMI ≥ 30 was independently associated with higher EBL and longer operative time, and prior surgery also prolonged operative time. Surgeon effects were pronounced for efficiency and nodal retrieval; BMI was not associated with lymph-node yield. LOS was not associated with BMI; however, LOS was higher in later surgical years and varied by surgeon.

**Conclusion:**

In standardized robot-assisted endometrial cancer surgery, obesity increased intraoperative workload (blood loss and operative time) without reducing lymph-node yield. LOS reflected surgeon- and time-related factors rather than BMI. These findings support the feasibility of robotic surgery in patients with obesity and underscore the importance of surgeon experience, pathway fidelity, and ongoing quality improvement.

## Highlights

• Obesity independently increased blood loss and operative time in robotic surgery.
• BMI ≥ 30 kg/m² did not compromise pelvic lymph-node yield.
• Length of stay was influenced by surgeon and surgical era, not BMI.
• Standardized robotic surgery was feasible and oncologically adequate in obesity.

## 1 Introduction

The prevalence of endometrial cancer is increasing worldwide possibly because of the aging society and higher rates of obesity [1]. According to randomized trials and subsequent meta-analyses, minimally invasive surgery (MIS) offers comparable oncologic outcomes to those of laparotomy while providing clear perioperative advantages [2], [3]. In particular, MIS is associated with reduced blood loss and shorter hospital stays compared with open surgery [4]. Consequently, it is recommended by major European guidelines as the preferred surgical staging approach for endometrial cancer [5], [6].

Robotic surgery (RS) further enhances MIS through three-dimensional visualization, articulated instruments with greater precision, improved ergonomics, and reduced surgeon fatigue and tremor [7]. In particular, these technical advantages may be beneficial in patients with elevated body mass index (BMI), who often present with increased technical complexity. However, the specific effects of obesity on perioperative outcomes in RS remain unknown, with existing studies demonstrating heterogeneous methodologies and inconsistent results [8], [9]. Importantly, no completed randomized controlled trials have specifically evaluated the perioperative effects of RS in cohorts restricted to obese patients. Therefore, high-level evidence in this population remains limited. Moreover, considering the substantial heterogeneity in surgical procedures, perioperative pathways, and institutional practices across published cohorts, the reliability and generalizability of these findings are limited [8], [10]. Furthermore, surgeon-related factors and institution-specific pathways can independently influence operative efficiency and postoperative recovery, complicating the attribution of perioperative outcomes solely to BMI [11], [12].

To address these research gaps, the present study investigated whether BMI ≥ 30 kg/m² independently affects estimated blood loss (EBL), operative time, length of stay (LOS), and lymph-node yield in patients undergoing a standardized robotic procedure (total hysterectomy with bilateral salpingo-oophorectomy and pelvic lymphadenectomy) at a single institution. Multivariable analyses were performed adjusting for key clinical and procedural covariates, including age, previous surgical history, comorbidities, operating surgeon, and year of surgery. Given the limited number of obese patients and the retrospective nature of the study, this analysis was designed as an exploratory investigation, and non-significant findings were interpreted with caution.

## 2 Methods

### 2.1 Study design and setting

This retrospective, single-center cohort study was performed at Dokkyo Medical University Saitama Medical Center. All consecutive patients with endometrial cancer who underwent robot-assisted total hysterectomy with bilateral salpingo-oophorectomy and pelvic lymphadenectomy using the da Vinci Xi system (Intuitive Surgical, Sunnyvale, CA, USA) between November 2018 and June 2025 were included. All procedures were performed by three attending surgeons following a standardized operative protocol.

### 2.2 Data collection

Data, including age, BMI, parity, comorbidities, history of abdominal/pelvic surgery, operative time, intraoperative EBL, lymph-node yield, LOS from surgery to discharge, final histopathology, and FIGO 2008 stage, were collected from electronic health records. EBL was recorded based on standardized anesthetic records, incorporating suction volume and gauze weight estimation routinely used at our institution. LOS was defined as the number of postoperative days from surgery to hospital discharge.

### 2.3 Statistical analysis

Continuous variables were summarized as medians with interquartile range and compared between BMI groups using Wilcoxon’s rank sum test. Meanwhile, categorical variables were expressed as frequencies and percentages and compared using the chi-square test or Fisher’s exact test, where appropriate. Multivariable linear regression models were constructed to estimate the independent effect of BMI ≥ 30 kg/m² for the primary outcome measures, including EBL, operative time, lymph-node yield, and LOS. Each model was adjusted for clinically relevant covariates, including age (continuous, in years); previous abdominal or pelvic surgery (yes/no); comorbidity history defined as the presence of diabetes, hypertension, or dyslipidemia (yes/no); operating surgeon (three categories: Surgeon A, Surgeon B, and Surgeon C); and surgical year dichotomized into an early period (2018–2020) versus a later period (2021–2025) to account for institutional learning curve and changes in perioperative protocols. The reference categories included BMI < 30 kg/m², no previous surgery, no comorbidity history, Surgeon C, and 2018–2020 period. Regression coefficients with 95 % confidence intervals (CIs) were calculated to quantify the magnitude and precision of each covariate’s association with the outcome. Model assumptions were verified by examining residual plots and assessing multicollinearity using variance inflation factors. Although some outcome variables demonstrated right-skewed distributions, linear regression was selected for interpretability given the modest sample size and exploratory aim of the study. All statistical analyses were performed using STATA 18.0 (StataCorp, College Station, TX, USA), and statistical significance was considered at *P* < 0.05.

## 3 Results

### 3.1 Patient characteristics

This study included 119 patients, who were categorized by BMI as follows: BMI ≥ 30 kg/m² (n = 32) and BMI < 30 kg/m² (n = 87). The median age of patients was 51.5 years (range, 24–67 years) in the BMI ≥ 30 group and 56 years (range, 34–80 years) in the BMI < 30 group, with a statistically significant difference (*p* = 0.006). The median parity was 1 (range 0–4) in the BMI ≥ 30 group and 2 (range 0–3) in the BMI < 30 group, with no significant difference (*p* = 0.35). In terms of comorbidities, diabetes was more prevalent in the BMI ≥ 30 group (31.2 %, 10/32) than in the BMI < 30 group (9.2 %, 8/87; *p* = 0.007). Meanwhile, the prevalence of hyperlipidemia was similar between both groups (12.5 % vs. 11.5 %; *p* = 1.0). Hypertension was significantly more common in the BMI ≥ 30 group than in the BMI < 30 group (43.8 % vs. 20.7 %; *p* = 0.019). The incidence of other comorbidities (21.9 % vs. 19.5 %; *p* = 0.80) and previous abdominal or pelvic surgery (43.8 % vs. 40.2 %; *p* = 0.83) was comparable between both groups. The distribution of cases by surgeon was as follows: Surgeon C managed 63/87 (72.4 %) and 22/32 (68.8 %) patients in the BMI < 30 and ≥ 30 groups, Surgeon A managed 17/87 (19.5 %) and 6/32 (18.8 %) patients in the BMI < 30 and ≥ 30 groups, and Surgeon B managed 7/87 (8.0 %) and 4/32 (12.5 %) patients in the BMI < 30 and ≥ 30 groups, with no significant difference (*p* = 0.74). The disease stage was comparable between groups, with stage IA being observed in 84.4 % and 74.7 % of patients in the BMI ≥ 30 and < 30 groups, respectively (*p* = 0.33). Histopathological analysis predominantly revealed endometrioid type, with grade 1 being observed in 90.6 % and 83.9 % of patients in the BMI ≥ 30 and < 30 groups, respectively (*p* = 0.50) (Table 1).

**Table 1:** Baseline characteristics by BMI group.

**Characteristic**		**BMI < 30 (n = 87)**	**BMI ≥ 30 (n = 32)**	**p-value**
Age, years (median [IQR])		56 (50–62)	51.5 (43–57)	0.006
Parity (median [IQR])		2 (0–2)	1 (0–2)	0.35
Comorbidities				
	Diabetes, n (%)	8 (9.2 %)	10 (31.2 %)	0.007
	Hyperlipidemia, n (%)	10 (11.5 %)	4 (12.5 %)	1.0
	Hypertension, n (%)	18 (20.7 %)	14 (43.8 %)	0.019
	Other comorbidities, n (%)	17 (19.5 %)	7 (21.9 %)	0.80
Prior abdominal/pelvic surgery, n (%)		35 (40.2 %)	14 (43.8 %)	0.83
Operating surgeon				0.74
	A, n (%)	17 (19.5 %)	6 (18.8 %)	
	B, n (%)	7 (8.0 %)	4 (12.5 %)	
	C, n (%)	63 (72.4 %)	22 (68.8 %)	
FIGO stage				0.33
	IA, n (%)	65 (74.7 %)	27 (84.4 %)	
	IB, n (%)	15 (17.2 %)	2 (6.2 %)	
	II, n (%)	2 (2.3 %)	1 (3.1 %)	
	IIIA, n (%)	1 (1.1 %)	0 (0.0 %)	
	IIIC1, n (%)	4 (4.6 %)	2 (6.2 %)	
Histology				
	Endometrioid G1, n (%)	73 (83.9 %)	29 (90.6 %)	0.50
	Endometrioid G2, n (%)	12 (13.8 %)	2 (6.2 %)	
	Endometrioid G3, n (%)	1 (1.1 %)	1 (3.1 %)	
	SEIC, n (%)	1 (1.1 %)	0 (0.0 %)	

BMI, Body Mass Index; IQR, interquartile range; FIGO, International Federation of Gynecology and Obstetrics; SEIC, Serous Endometrial Intraepithelial Carcinoma

### 3.2 Outcome

#### 3.2.1 EBL

Multivariable linear regression analysis revealed that BMI ≥ 30 kg/m² was independently associated with higher EBL (coefficient 51.47 mL; 95 % CI 9.52–93.41; *p* = 0.016). Age (−0.66 mL per year; 95 % CI −2.50–1.18; *p* = 0.47), previous surgery (−7.83 mL; 95 % CI −44.32–28.66; *p* = 0.67), comorbidity history (−18.07 mL; 95 % CI −54.89–18.75; *p* = 0.33), surgeon (e.g., Surgeon B −29.22 mL; 95 % CI −91.52–33.08; *p* = 0.35), and surgical year (2021–2025 vs 2018–2020: −23.63 mL; 95 % CI −64.23–16.96; *p* = 0.25) were not significant (Table 2).

**Table 2:** Multivariable model: estimated blood loss.

**Variables**	**Coefficient (mL)**	**SE**	**β**	**95 %CI**	**P value**
BMI ≥ 30 (vs <30)	51.47	21.16	0.2392	9.52–93.41	0.016
Age (per year)	-0.66	0.93	-0.0721	-2.50–1.18	0.47
Prior surgery (yes vs no)	-7.83	18.41	-0.0404	-44.32–28.66	0.67
Comorbidity history (yes vs no)	-18.07	18.58	-0.0947	-54.89–18.75	0.33
Surgeon A (vs reference: C)	-17.23	23.34	-0.0713	-63.49–29.04	0.46
Surgeon B (vs reference: C)	-29.22	31.44	-0.0887	-91.52–33.08	0.35
Surgical year 2021–2025 (vs 2018–2020)	-23.63	20.48	-0.1163	-64.23–16.96	0.25

BMI, Body mass index; SE, Standard error; CI, Confidence interval

#### 3.2.2 Operative time

BMI ≥ 30 kg/m² (34.97 min; 95 % CI 7.64–62.29; *p* = 0.012) and previous surgery (26.50 min; 95 % CI 2.72–50.28; *p* = 0.029) were associated with a longer operative time. Pronounced surgeon effects were observed (e.g., Surgeon B: −95.20 min; 95 % CI −135.79 to −51.61; *P* < 0.001; Surgeon A: −40.88 min, CI as shown in tables; *p* = 0.008). Age (−0.27 min per year; 95 % CI −1.48–0.92; *p* = 0.64), comorbidity history (2.20 min; 95 % CI −21.79–26.19; *p* = 0.85), and surgical year (11.95 min; 95 % CI −14.49–38.40; *p* = 0.37) were not significant (Table 3).

**Table 3:** Multivariable model: operative time.

**Variables**	**Coefficient (min)**	**SE**	**β**	**95 %CI**	**P value**
BMI ≥ 30 (vs <30)	34.97	13.79	0.2255	7.64–62.29	0.012
Age (per year)	-0.27	0.66	-0.042	-1.48–0.92	0.64
Prior surgery (yes vs no)	26.5	12.00	0.1897	2.72–50.28	0.029
Comorbidity history (yes vs no)	2.2	12.1	0.0159	-21.79–26.19	0.85
Surgeon A (vs reference: C)	-40.88	15.21	-0.2348	-71.02 to −15.21	0.008
Surgeon B (vs reference: C)	-95.2	20.48	-0.401	-135.79 to −51.61	< 0.001
Surgical year 2021–2025 (vs 2018–2020)	11.95	13.34	0.0816	-14.49–38.40	0.37

BMI, Body mass index; SE, Standard error; CI, Confidence interval

#### 3.2.3 Lymph-node yield

BMI ≥ 30 kg/m² showed no association with lymph-node yield (5.10 nodes; 95 % CI −0.25–10.46; *p* = 0.061). However, surgeon effects were significant (e.g., Surgeon A: −13.09 nodes; 95 % CI −19.00 to −7.18; *p* < 0.001; Surgeon B: −9.99 nodes; 95 % CI −17.95 to −2.03; *p* = 0.014). Previous surgery (0.27 nodes; 95 % CI −4.39–4.93; *p* = 0.90), comorbidity history (3.46 nodes; 95 % CI −1.23–8.17; *p* = 0.14), and surgical year (−0.08 nodes; 95 % CI −5.26–5.10; *p* = 0.97) were not significant (Table 4).

**Table 4:** Multivariable model: lymph-node yield.

**Variables**	**Coefficient (nodes)**	**SE**	**β**	**95 %CI**	**P value**
BMI ≥ 30 (vs <30)	5.1	2.70	0.1714	-0.25–10.46	0.061
Age (per year)	-0.13	0.11	-0.1095	-0.37–0.09	0.24
Prior surgery (yes vs no)	0.27	2.35	0.0101	-4.39–4.93	0.9
Comorbidity history (yes vs no)	3.46	2.37	0.1312	-1.23–8.17	0.14
Surgeon A (vs reference: C)	-13.09	2.98	-0.3917	-19.00 to −7.18	< 0.001
Surgeon B (vs reference: C)	-9.99	4.01	-0.2192	-17.95 to −2.03	0.014
Surgical year 2021–2025 (vs 2018–2020)	-0.08	2.61	-0.0029	-5.26–5.10	0.97

BMI, Body mass index; SE, Standard error; CI, Confidence interval

#### 3.2.4 LOS

BMI ≥ 30 kg/m² was not associated with LOS (0.93 days; 95 % CI −0.61–2.48; *p* = 0.23). However, LOS was shorter for Surgeon B (−3.18 days; 95 % CI −5.48 to −0.89; *p* = 0.006), but the difference was not significant for Surgeon A (−1.10 days; 95 % CI −2.81–0.60; *p* = 0.20). Comorbidity history was also not significant (−0.10 days; 95 % CI −1.46–1.25; *p* = 0.87). Later surgical years were associated with longer LOS (1.93 days; 95 % CI 0.43–3.43; *p* = 0.011) (Table 5).

**Table 5:** Multivariable model: length of stay (surgery to discharge).

**Variables**	**Coefficient (days)**	**SE**	**β**	**95 %CI**	**P value**
BMI ≥ 30 (vs <30)	0.93	0.78	0.1173	-0.61–2.48	0.23
Age (per year)	-0.01	0.34	-0.0360	-0.08–0.05	0.72
Prior surgery (yes vs no)	-0.14	0.67	-0.0206	-1.49–1.19	0.82
Comorbidity history (yes vs no)	-0.10	0.68	-0.0153	-1.46–1.25	0.87
Surgeon A (vs reference: C)	-1.1	0.86	-0.1232	-2.81–0.60	0.20
Surgeon B (vs reference: C)	-3.18	1.16	-0.2611	-5.48 to −0.89	0.006
Surgical year 2021–2025 (vs 2018–2020)	1.93	0.75	0.2569	0.43–3.43	0.011

BMI, Body mass index; SE, Standard error; CI, Confidence interval

## 4 Discussion

### 4.1 Principal findings

In this single-center cohort of 119 patients undergoing a standardized robotic approach for endometrial cancer, obesity (BMI ≥30 kg/m²) was independently associated with increased EBL and prolonged operative time. By contrast, BMI was not associated with lymph-node yield. Meanwhile, the LOS varied according to the operating surgeon and increased in later surgical years. These associations remained robust after adjusting for age, history of previous abdominal or pelvic surgery, comorbidity history, operating surgeon, and surgical year, underscoring the independent contribution of BMI to intraoperative workload. However, the magnitude of these associations was modest and should be interpreted in the context of the study’s exploratory design.

### 4.2 Comparison with literature

According to meta-analyses and large comparative cohorts, MIS, including robotic and conventional laparoscopic approaches, is associated with reduced blood loss and shorter hospitalization compared with laparotomy, without compromising short-term oncologic outcomes. Although the magnitude and consistency of perioperative benefits vary across studies and patient subgroups, these advantages are generally maintained in obese and morbidly obese populations, being supported most robustly by large network meta-analyses and selected comparative cohorts [9], [13], [14], [15].

In obese patients, the LOS can generally be maintained within standardized perioperative care pathways. In parallel, contemporary perioperative management increasingly incorporates standardized pathways, including enhanced recovery after surgery (ERAS), a multimodal, evidence-based approach designed to attenuate surgical stress responses, accelerate functional recovery, and reduce postoperative morbidity [16]. However, previous studies reported increased EBL and longer operative times with increasing BMI, a trend commonly attributed to technical challenges (e.g., limited access through a thicker abdominal wall, restricted working angles, and prolonged instrument manipulation) [14], [15], [17].

Furthermore, based on systematic reviews focusing on obese populations, laparoscopic and robotic approaches achieve low conversion rates and acceptable complication profiles. Although RS has demonstrated comparable or, in some series, lower conversion rates compared with laparoscopy in higher-BMI cohorts, the substantial heterogeneity across studies highlights the complexity of interpreting these findings and precludes definitive conclusions regarding superiority [8], [18].

Comparative and randomized data also suggest that robotic platforms may partly mitigate visualization and ergonomic constraints associated with conventional laparoscopy in patients with challenging body habitus, albeit sometimes at the cost of longer operative times. These findings are supported by comparative studies and randomized cohorts, including those with available medium-term follow-up. However, the available evidence remains limited by heterogeneity in study design and outcome reporting [19], [20].

Provider-related factors also play an equally important role. Learning-curve analyses consistently demonstrate reductions in operative time with increasing case volume, emphasizing the impact of surgeon experience, coordinated team workflow, and familiarity with the robotic platform on perioperative efficiency [21], [22].

With regard to oncologic completeness, existing evidence does not support a consistent BMI-related reduction in lymph-node yield during robotic staging procedures. Instead, surgical strategy, technical execution, and pathological handling appear to exert a more decisive influence [23], [24].

Beyond BMI, emerging data indicate that computed tomography-derived body composition parameters, particularly visceral adipose tissue burden, may better capture technical complexity and provide more refined perioperative risk stratification in endometrial cancer surgery [25], [26].

Collectively, the literature supports a nuanced interpretation: obesity is associated with increased intraoperative effort, as indicated by longer operative times and moderately higher blood loss. However, nodal assessment and postoperative recovery are not necessarily compromised when robotic-assisted MIS is delivered within standardized pathways (e.g., ERAS) by experienced teams, highlighting the importance of structured perioperative management and institutional expertise in mitigating BMI-related operative burden [15], [20], [24], [27]. Importantly, obesity has also been associated with potential negative perioperative effects in prior reports, including prolonged operative time, increased blood loss, and higher complication risk, particularly in patients with extreme obesity. Although such adverse outcomes were not consistently observed in the present cohort, these risks should be acknowledged when interpreting the findings and counseling patients.

### 4.3 Strengths

A major strength of this study is the multivariable framework explicitly adjusting for the operating surgeon and surgical year in addition to patient-level covariates. This approach reduces attribution bias when estimating BMI’s independent effect and allows for differentiation between patient habitus and influence of operator skill and institutional maturation—factors that are frequently unaccounted for in observational studies.

### 4.4 Limitations and potential biases

Despite the strength, this study has several limitations. Its retrospective design introduces a risk of selection bias as case allocation and perioperative decision-making may have differed by surgeon or time period. If more complex cases were preferentially assigned to certain operators or eras, BMI’s estimated effect may have been under- or overestimated, despite adjustment. In addition, although surgeon was included as a covariate, clustering of outcomes within surgeons could not be fully accounted for because of the limited number of surgeons and events.

Information and measurement bias may also be present. EBL was based on clinical estimation rather than objective measurement. Moreover, the lymph-node yield might have been influenced by variation in pathological processing and specimen submission practices. In addition, residual confounding cannot be excluded as some variables (e.g., uterine size, detailed body composition metrics, anesthetic techniques, assistant experience, and timing and fidelity of ERAS implementation) were not captured, which might have affected the operative time and LOS. Additionally, obesity was dichotomized using a BMI cutoff of 30 kg/m², and more granular analyses across obesity classes were not feasible because of sample size limitations.

Furthermore, the study was underpowered to evaluate rare outcomes. Only one case required conversion to laparotomy, and perioperative complications were infrequent, precluding meaningful statistical analysis of these endpoints.

Finally, generalizability was limited by the single-center design, and precision was reduced in the BMI ≥ 30 group, which yielded wider CIs for some outcomes.

### 4.5 Future directions

Prospective multicenter studies incorporating standardized ERAS protocols, granular performance metrics (e.g., surgeon- and assistant-specific experience), and cross-sectional imaging for body composition analysis may enable more precise risk stratification beyond BMI and help identify modifiable workflow targets. In addition, time–motion analyses and platform-specific benchmarks could further translate surgeon- and year-related effects into reproducible quality-improvement strategies [22], [23].

In parallel**,** a randomized controlled trial evaluating robotic-assisted versus conventional laparoscopic surgery in obese patients with early endometrial cancer in the sentinel lymph node era (RObese) is currently ongoing [28]. The results of this study are awaited and may further clarify the comparative role of these approaches in the surgical management of obese patients.

## 5 Conclusion

Within a standardized robotic surgical approach for endometrial cancer, obesity was independently associated with statistically significant increases in operative time and EBL. However, LOS and lymph-node yield were more strongly influenced by surgeon-related and institutional maturation factors. These findings support the technical feasibility of RS in obese patients within experienced centers, and highlight the need for optimized perioperative planning and continued refinement of team-based workflows to mitigate BMI-related operative burden.

## CRediT authorship contribution statement

**Yasushi Iida:** Writing – review & editing, Writing – original draft, Project administration, Methodology, Investigation, Data curation, Conceptualization. **Kosuge Ayane:** Investigation, Data curation. **Miwako Shimazaki:** Writing – original draft, Investigation, Data curation. **Kosuke Kato:** Writing – review & editing. **Takahiro Matsunami:** Writing – review & editing. **Teppei Ichikawa:** Writing – review & editing. **Makoto Iizuka:** Writing – review & editing. **Taichi Irie:** Writing – review & editing. **Satoshi Takakura:** Writing – review & editing, Validation, Supervision. **Daishi Hirano:** Writing – review & editing, Formal analysis.

## Ethics approval and consent to participate

This retrospective study was approved by the Institutional Review Board of Dokkyo Medical University Saitama Medical Center (Approval No. 25056). The requirement for individual informed consent was waived owing to the retrospective design and use of de-identified data, with an opt-out opportunity provided on the institutional website in accordance with institutional policy and the Declaration of Helsinki.

## Funding

This research did not receive any specific grant from funding agencies in the public, commercial, or not-for-profit sectors.

## Declaration of Competing Interest

The authors declare that they have no known competing financial interests or personal relationships that could have appeared to influence the work reported in this paper.

## Data Availability

De-identified data underlying this article are available from the corresponding author on reasonable request.
